# Re: Patel et al: Oral Antithrombotic Medication Is Associated with Improved Visual Acuity Outcomes in Eyes with Neovascular Age-Related Macular Degeneration

**DOI:** 10.1016/j.xops.2025.100840

**Published:** 2025-06-03

**Authors:** Pierre-Henry Gabrielle, Catherine Creuzot-Garcher

**Affiliations:** Ophthalmology Department, Dijon University Hospital, Dijon, France

TO THE EDITOR:

We read with interest the article by Patel et al.^1^ The authors conclude that oral anticoagulants (AC) were associated with improved long-term visual outcomes in patients with submacular hemorrhage (SMH) secondary to neovascular age-related macular degeneration. However, we believe the interpretation of their results overstates the therapeutic implication of antithrombotic use, and respectfully raise 3 key methodological concerns.

First, while the authors correctly adjusted their models for baseline visual acuity (VA), they focused on the gain in VA rather than on the final VA itself. This analytical choice can overemphasize improvement, especially when baseline VA differs between groups, even after statistical adjustment. Notably, the final crude VA was not significantly better in the AC group (20/250 vs. 20/750; *P* = 0.11). Hence, the main conclusion suggesting a visual benefit from AC use remains unsupported by the most clinically relevant end point. Analyses should combine final-adjusted VA and visual gain or prioritize final outcome models rather than change-from-baseline scores, which are more susceptible to error and misinterpretation.^2^

Second, although the authors included treatment modality as a covariate in their models (anti-VEGF monotherapy, pneumatic displacement, or vitrectomy), the effectiveness of SMH displacement on visual outcomes in SMH is well established and potentially nonlinear. The Surgery, Tissue Plasminogen Activator, Antiangiogenic Agents, and Age-Related Macular Degeneration (STAR) trial demonstrated that SMH displacement by pneumatic displacement or vitrectomy combined with anti-VEGF injection was associated with excellent visual gains, which were not previously achieved using anti-VEGF monotherapy.^3^ However, the distribution of these treatments across the AC exposure groups was not reported. This omission is critical: if patients on ACs disproportionately received more effective local therapies, such as vitrectomy or pneumatic displacement, this could explain their better visual outcomes. Without this information, the reader cannot determine whether the observed association reflects a true effect of anticoagulation or was confounded by differences in retinal treatment allocation. A stratified analysis or interaction modeling would be necessary to clarify this point.

Third, the article does not report how ACs were managed at the time of SMH and after SMH. Even though it has been shown that continuing ACs at the time of vitreoretinal surgery does not increase bleeding complications,^4^^,^^5^ the clinical decision to stop or maintain these drugs could directly impact outcomes and how the authors interpreted results. This information is essential and should have been adjusted for and reported.

In light of these considerations, the current data do not support the conclusion that oral antithrombotic medication improved visual prognosis in SMH-complicating neovascular age-related macular degeneration. Instead, they might reflect residual confounding and overinterpretation of VA gains without a difference in final visual outcomes.

Given the absence of clear information on SMH displacement treatment allocation and antithrombotic management and a significant difference in final visual outcomes, we believe the association between antithrombotic medication use and improved vision in SMH should be interpreted cautiously.

Sincerely,
